# Prevalence of physiological and perceptual markers of low energy availability in male academy football players: a study protocol for a cross-sectional study

**DOI:** 10.1136/bmjsem-2024-002250

**Published:** 2024-10-07

**Authors:** Jamie Ashby, Thomas Mullen, Philip Smith, John P Rogers, Nick Dobbin

**Affiliations:** 1 Department of Health Professions, Faculty of Health and Education, Manchester Metropolitan University, Manchester, UK; 2 Department of Sport and Exercise Sciences, Manchester Metropolitan University Institute of Sport, Manchester, UK; 3 The OrthTeam Centre, Manchester, UK

**Keywords:** Football, Nutrition, Adolescent, Sports & exercise medicine, Injury

## Abstract

Low energy availability (LEA) is a core feature of the female athlete triad and relative energy deficiency in sport (REDs). LEA underpins multiple adverse health and performance outcomes in various athletic populations, including weight category, endurance and aesthetic sports. Recent reports suggest LEA is highly prevalent in female football, volleyball and netball, with little known on male team-sport athletes. Therefore, the study aims to identify the prevalence of LEA among male academy football players (16–23 years), using surrogate markers that align with the International Olympic Committee REDs Clinical Assessment Tool-Version 2. A cross-sectional study design will be used with physiological and perceptual markers of LEA measured. The study will seek to recruit 355 players to complete several online questionnaires believed to be associated with LEA, measured using a 24-hour food and activity diary. Of the 355 players, a subsample (n=110) will complete an additional 3-day food and activity diary, provide a venous blood sample to measure levels of total testosterone and free triiodothyronine, and have resting metabolic rate (RMR) measured to determine RMR_ratio_. The prevalence of LEA will be determined using the low (<30 kcal·kgFFM^-1^·day^-1^) domain of energy availability and divided by the total number of participants. Descriptive statistics will be used to summarise the whole group and difference status of energy availability (eg, low, reduced, optimal, high). A univariable and multivariable binary logistic regression analysis will be modelled to assess the association of various surrogate markers with the presence of LEA.

**WHAT IS ALREADY KNOWN ON THIS TOPIC**

**WHAT THIS STUDY ADDS**

- Using a multiclub approach, this will be the first study to determine the prevalence of physiological and perceptual markers of LEA in male academy football players in the UK.
- This study will explore the association of various physiological and perceptual markers and LEA, providing a better understanding of the ‘tools’ available to clinicians and practitioners.

**HOW THIS STUDY AFFECTS RESEARCH, PRACTICE, OR POLICY**

- The findings of this study could support the application of preventative and screening methods for LEA suitable for young male team-sport athletes. Thus, this study’s findings will help shape future sports medicine and nutrition practices within football academies.

## Introduction

 Low energy availability (LEA) is an imbalance between energy intake and exercise energy expenditure, taking into consideration fat free mass (FFM).^1^ A state of LEA can occur when an athlete fails to meet energy intake recommendations, resulting from poor nutritional knowledge, insufficient food preparation skills or limited access to high-quality foodstuff. Conversely, LEA could occur from athletes intentionally restricting energy intake combined with compulsive training practices to manipulate body composition. Without careful consideration, this may lead to disordered eating behaviours or potentially an eating disorder diagnosis.^2^ Regardless of the scenario, reduced energy availability will lead to a repartitioning of fuel away from reproduction and growth to sustain the immediate physical demands.^3^ A chronic state of energy deficiency is thought to impair physiological adaptation and recovery, increase injury risk, suppress immune function, increase the risk of burnout and, in prolonged extreme cases, lead to long-term health complications such as osteoporosis.^1^ A state of LEA is associated with hormonal fluctuations in males, with evidence suggesting that testosterone levels become suppressed after just 5 days of LEA.^4^ Elsewhere, Stenqvist *et al*^5^ observed a reduction in the resting metabolic rate (RMR_ratio_) (−3.3%), insulin (−11.0%), IGF-1 (−1.0%) and triiodothyronine (−5.0%) following 4 weeks of intensified training, while habitual exercise and dietary intake were maintained. Importantly, performance outcomes are not fully restored in athletes with 2 days of an optimal energy balance following 10 days of LEA.^6^ Such changes demonstrate the necessity to periodise and manage dietary intake according to training volume and intensity to avoid LEA, which many athletes cannot do in practice.^7^ Given the limited research on LEA within male athletes and the difficulty of accurately measuring energy availability, surrogate markers are needed to help identify LEA within this population.^8^

Within the literature, research on LEA has mostly focused on female populations^9^ or those competing in weight categories, endurance or aesthetic sports.^10^,^12^ The focus on these athletic groups is understandable given they are widely recognised as ‘high risk’ of LEA. In part, this is due to several factors, including high training volumes, pressure of ‘making’ weight, and a greater prevalence of disordered eating and/or eating disorders.^3^ As such, body image issues can arise due to various internal and external pressures (eg, social media, selection, judges scoring, sports commentators),^12 13^ leading an athlete to unhealthy nutritional and/or training practices, which results in LEA.

Given the implications of LEA, and with recent research starting to observe the consequences of LEA in male team sport populations,^14 15^ there is a need to conduct sport-specific research to establish the prevalence of LEA, given the unique nutritional requirements for individual sporting disciplines.^16^ One group that warrants further consideration is male academy football players, where it has been demonstrated that energy and carbohydrate periodisation does not occur^7 17^ and the total energy expenditure is higher than age-matched non-academy players.^18^ Furthermore, players’ RMR increases with age^19^ and changes across the competitive match week.^7^ Data collected recently by two authors of this protocol (unpublished), using a 3-day food diary, RMR-adjusted metabolic equivalent (MET) values^20^ and an activity diary, revealed that 45% of academy footballers were in a state of LEA (<30 kcal·kgFFM^-1^·day^-1^) on all 3 days of the observation period. Such findings are concerning given that these individuals are undergoing key stages of maturation (eg, changes in stature, body mass (BM), FFM and metabolic potential)^19^ in conjunction with an increased training load and injury risk.^21^ Hence, practitioners (eg, sports medicine practitioners, nutritionists, sports scientists and coaches) must work collaboratively to ensure optimal energy availability to maintain health, fuel growth, and maximise athletic performance in young male football players.

The purpose of this protocol is to describe the research question, aims, objectives and methodological approach adopted in a cross-sectional study investigating the prevalence of physiological and perceptual markers of LEA in male academy football players.

### Research question, aim and objectives

The research question for the cross-sectional study is: What is the prevalence of physiological and perceptual markers of LEA within male academy football players?

The study will aim to identify the prevalence of LEA among male academy football players (16–23 years), using surrogate markers that align with the International Olympic Committee REDs Clinical Assessment Tool-Version 2.

To answer the research question, we have set out to meet the following objectives:

1. To estimate energy intake and expenditure to calculate energy availability for the entire sample.
2. To categorise energy availability status into four domains (high, optimal, reduced and low) for the entire sample.
3. To determine if an association exists between the sample’s responses to perceptual measures and LEA for the entire sample.
4. To determine if an association exists between physiological markers and LEA for the subgroup only.

## Methods

### Study design

The study will use a cross-sectional design to capture quantitative data on surrogate markers of LEA in male academy football players. The study will be guided by the STrengthening the Reporting of OBservational studies in Epidemiology (STROBE) guidelines.^22^

**Figure 1:**
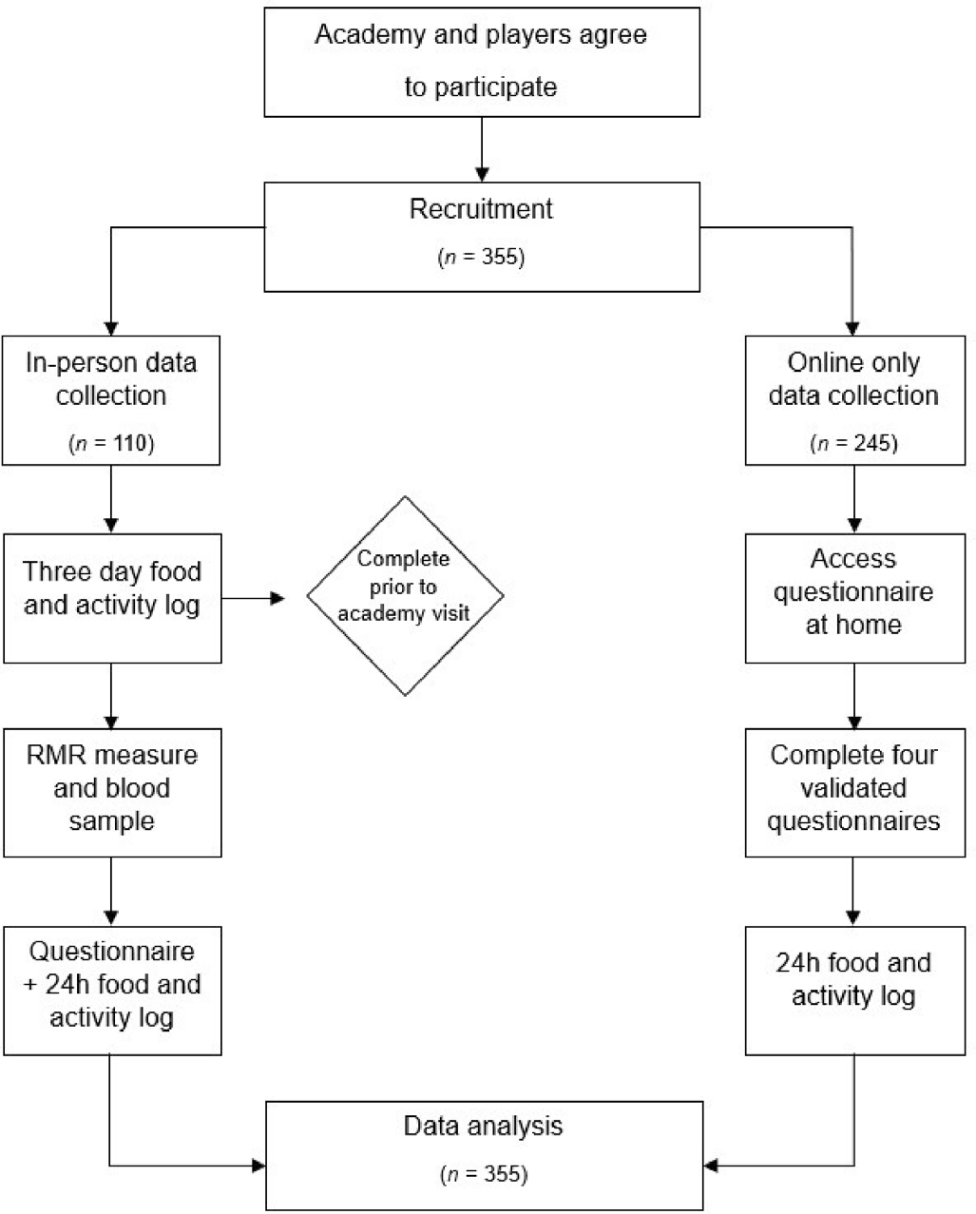
A visual representation of the participant’s journey for each arm of the study. RMR, resting metabolic rate; n, number of participants.

The study will adopt two parallel pathways to achieve the objectives feasibly and pragmatically. All participants (objectives 1, 2 and 3) will complete an online questionnaire assessing components of, or factors associated with, LEA, built into a JISC survey (Jisc Services Limited, Bristol, UK). In addition, a subgroup of participants will complete the questionnaire in person, an assessment of RMR, and provide a blood sample (figure 1). The subgroup data collection will take place on-site at each participating football academy.

### Eligibility criteria

The participants must be able to read and write in English and be affiliated with a football academy in the UK to participate in the study. Participants currently undergoing treatment for an eating disorder or disordered eating will be excluded from the study to minimise any interference and risk. The club will be asked for known cases within the targeted age groups. Participants will also be excluded if they are not training due to injury or missed ≥50% of their training sessions the previous week.

### Sample size

Objectives 1 and 2 concern estimating energy availability and the prevalence of LEA within academy football players. To determine this, the current estimated prevalence was taken from Scheffer *et al*^23^ at 64% with a margin of error of 5% and a 95% CI. Using these values within the EpiTool calculator,^24^ a sample of ~355 is required for adequate precision of the estimate.

Objectives 3 and 4 aim to understand the value of RMR_ratio_, total testosterone and free triiodothyronine (T_3_) as markers of LEA and will be collected alongside the online questionnaire (ie, low/normal sex drive, low/high burnout, low/high training distress and low/high risk of eating disorder). A binary logistic regression will determine the association between categorised data (eg, low/normal T_3_) and LEA using a 30 kcal·kgFFM^-1^·day^-1^ cut-off. There is minimal research on LEA in male academy football players. Therefore, a sample size estimation was derived in G*Power based on previous prevalence rates of 64%.^23^ A two-tailed test will be used to reflect differing associations. The alpha level is set to 0.05, and the power at 0.8. ‘R^2^ other X’ is set at 0 as the shared variance between the measures is currently unknown. The X distribution was set to binomial with <30 kcal·kgFFM^-1^·day^-1^ and >30 kcal·kgFFM^-1^·day^-1^ used. The estimated sample size for in-person data collection is 110 to determine associations between physiological and perceptual markers and LEA.

### Recruitment

Convenience sampling will be used for recruitment, and contacts known to the research team will be approached to gauge interest in the study. If they agree, the study’s preferred approach (online only vs in-person with direct measures) will be selected by the club until 110 participants have been achieved. Only after that will online participation be offered.

If the club prefer an online method, an information sheet will be shared with participants that contains information about the study, a video link to further information and a link to the questionnaire. Participants can then complete this questionnaire within a period agreed with the club. If the club choose the in-person method, the participants will be given an information sheet that contains a video link to further information but no questionnaire, as this will be accessed by participants on the day of data collection.

### Data collection

The questionnaire has been designed to include a non-validated section to ascertain key information about the participants (ie, name, age, height, weight, highest weight with current height, lowest weight with current height, diagnosis of chronic illness, treatment for any mental health symptoms within the last 12 months, food allergy, exclusion of food groups and history of bone stress injury; including the quantity and incidents within the previous 2 years and location) followed by multiple validated questionnaires (table 1) that are thought to be indicative of LEA.^1625^,^27^ The questionnaire responses will be transferred into a preformatted Microsoft Excel spreadsheet, which will provide an outcome for each validated questionnaire (ie, low/normal sex drive, low/high risk of burnout, low/high training distress, increased risk of an eating disorder) before being used for full analysis.

**Table 1:** Key information regarding the validated questionnaires to be administered to all participants

Questionnaire	Key information	Validated in population	Number of items	Justification	Cut-off scores
Low Energy Availability in Males Questionnaire(LEAM-Q)^16^	First questionnaire developed specifically for male athletes and energy availabilityDeveloped internationally with 310 athletes included in the final analysisParticipants answer four questions associated with sex drive	Male athletes across various weight-sensitive and non-weight-sensitive disciplines	4	Validated against clinical markers associated with LEAParticipants who scored as low sex drive demonstrated multiple perturbations to clinical factors associated with LEA compared with those that did not score a low sex driveRecommended for initial screening of LEA and RED-S^1^	Low sex drive was identified when ≥2 scored on item 1 or ≥2 scored on item 3 and ≥1 on item 4
Athlete Burnout Questionnaire(ABQ)^25^	Three domains scored separately to determine burnout (physical and emotional exhaustion, personal accomplishment and sport devaluation)Participants select one item on a 5-point scale (Almost never (1), Rarely (2), Sometimes (3), Frequently (4), Almost always (5))	Male and female adolescent athletes	15	Considered the ‘gold standard’ method of measuring burnout in athletesValidated for use with adolescent athletes in well-controlled laboratory and ecologically valid field-based conditions with athletes from various sporting disciplines	No clinically validated cut-off values to diagnose burnout, but an average of ≥3 across the domains may indicate potential burnout
The Training Distress Scale(TDS)^26^	Participants retrospectively rate (previous 48 hours) the intensity of 19 symptoms related to general fatigue on a 5-point scale (Not at all (0), A little bit (1), Moderate amount (2), Quite a bit (3), Extreme amount (4))Symptoms originally identified in a study of overtraining military recruits	Male and female triathletes and swimmers	19	Validated in well-controlled laboratory and ecologically valid field-based conditions showing dose-response sensitivity in both environments, with scores returning to baseline following reduction of training load	A total summed score of ≥12 appears to discriminate between a high or low training volume and would indicate that an athlete has experienced symptoms of physical and mental distress over the previous 48 hours
Brief Eating Disorder in Athletes Questionnaire(BEDA-Q)^27^	Participants provide a frequency of how often they agree to six statements on a 5-point scale (Never (1), Rarely (2), Sometimes (3), Usually (4), Always (5)). This is followed by 3 questions that ask about intentionally trying to lose weight (Yes or No) and the frequency of this (1–2, 3–5, >5)	Female adolescent athletes	9	Appears to distinguish between case and control well, with a sensitivity of 82.1% and specificity of 84.6%Validated for use in female athletes, but no items are female-specific	The total risk of an eating disorder is calculated on a continuous scale. A score of ≥27% indicates an athlete is at risk of an eating disorder

On the penultimate page of the questionnaire, all participants will complete a 24-hour retrospective food diary and activity log that reflects a ‘regular’ training day at the club. The participants will be asked to provide as much detail as possible relating to the type of food, time of consumption, meal composition, preparation method, addition of condiments, brand name and supplement use. The dietary intake data collected will be inputted into Nutritics software (Nutritics, Dublin, Ireland) by the lead researcher to determine energy and macronutrient intake. The activity log will be combined with an estimate or a directly measured value of RMR to calculate total energy expenditure. Hannon *et al*’s^19^ RMR equation, validated specifically for academy football players, will be used for the group completing the study online only. This will be measured directly for the subgroup, so both an estimate and a direct measure will be determined. Finally, to estimate energy availability, each player’s estimated lean BM (eLBM) will be calculated based on BM and stature, provided by the participants’ club, according to the Boer formula for men.^28^

### Sub-group

In addition to the questionnaires above, the subgroup will complete procedures to obtain data for additional physiological markers:

RMR will be measured via indirect calorimetry (Metalyzer 3B-R3, Cortex Biophysik GmbH, Leipzig, Germany) using an airtight mask and turbine (Hans Rudolph 7450, Shawnee, USA). Following calibration according to the manufacturer’s instructions, participants will lay in the supine position for 20 minutes and be instructed to remain still, avoid talking and not fall asleep. The test will be conducted in an overnight fasted state between 07:00 and 11:00 am in a quiet, dimly lit room.^29^ The first 10 min of measurement will be discarded, with the final 10 minutes used for analysis. A 30 s rolling average will be calculated and exported from the Cortex machine (MetaSoft Studio, Cortex Biophysik GmbH, Leipzig, Germany). The average value of VCO_2_ and VO_2_ (L/min) across the final 10 minutes will be used to determine the respiratory exchange ratio (RER) and energy equivalence. The data will be inspected for potential protocol violation, and a RER score of <0.7 and >1.0 will be removed from the analysis.^29^ Daily RMR (kcal·day^-1^) will be calculated from the energy equivalence and divided by estimated RMR (kcal·day^-1^)^19^ to determine RMR_ratio._ A score of <0.90 has demonstrated suppression of hormone function (low T_3_) and energy deficiency.^8^

Blood samples will be taken on-site at the football clubs involved in the in-person data collection to measure total testosterone and free T_3_ levels. The samples will be taken in an appropriate room at the club where the area can be sterilised before equipment is set out. All samples will be taken in an overnight fasted state between 07:00 and 11:00 am, immediately before or after the RMR measurement but not before at least 10 minutes of rest. In a seated position, one 10 mL sample will be taken from the medial cubital vein and placed in a vacutainer. The sample will be inverted 6 times and left to stand for 30 min. The serum will then be separated from whole blood using centrifugation (15 mins at 1500g). A pipette will transfer serum into an Eppendorf microtube and stored at −80°C in a non-HTA freeze, each labelled with a participant code. Serum samples will be analysed in duplicates to determine concentrations of total testosterone and free T_3_ using multiple ELISAs following the manufacturer’s instructions. The optical density of free T_3_ will be measured using a microplate reader at wavelengths 450 nm. Total testosterone will be measured at 405–420 nm. The output from the microplate will be inputted into a four-parameter logistic (4PL) curve whereby the optimal density of the sample is determined from the bottom plateau, top plateau, concentration of a half-maximal response, the Hill slope and concentration of the analyte from a standard.

The participants will be asked to record dietary intake across 3 days before data collection, including two training days and one rest day. The participants will use the Libro app to directly input dietary intake into the software via their smartphone, which will be accessible by the principal investigator on the desktop version. This method is anticipated to improve accuracy as the Libro app will provide portion quantities, typical household measures and supporting photos. Additionally, it will be completed on their phone in real-time, limiting recall bias. The participants will be provided with written and video instructions relating to the recording of food type, time of consumption, meal composition, preparation method, addition of condiments, brand name and supplement use. A mean value for each day of energy and macronutrient intake (g/kg BM) will be entered into a main data spreadsheet for each participant.

Across the same 3 days of recording dietary intake, the participants will record all purposeful exercises to estimate energy expenditure. This will be defined as any formal activity inside and/or outside their club, such as pitch-based, resistance and aerobic training. All entries will be submitted using the Libro app. The activity type and duration will be assigned a MET value from the compendium of physical activities^20^ and multiplied by their individual RMR (per minute).

### Statistical analysis

The estimated prevalence of LEA will be determined using the low (<30 kcal·kgFFM^-1^·day^-1^) domain of energy availability^1^ and divided by the total number of participants (n=355) to derive a point prevalence percentage.

Descriptive statistics will summarise each variable (see online supplemental appendix 1) and assumptions of normality will be assessed through visual inspection of the quantile-quantile plots. All data will be presented as a whole group and further categorised into different domains (low, reduced, optimal, high).

To determine the association between LEA (<30 kcal·kgFFM^-1^·day^-1^) and independent variables (suppressed RMR_ratio_ ≤0.9, low total testosterone ≤12 nmol/L, low free T_3_ ≤3.8 pmol/L, low sex drive, high burnout of ≥3 averaged across domains, high training distress ≥12, risk of eating disorder ≥27%), a binary logistic regression will be used where an OR >1 will indicate greater odds of LEA. The OR will be accompanied by 95% compatibility intervals. A univariable regression analysis will first be modelled to assess the association for each variable alone with energy availability (low vs reduced). Then, a multivariable regression analysis will be modelled with all variables to understand the model performance when adjusted for all predictors. Finally, a backward elimination will occur where variables with an OR between 0.77 and 1.30 will be excluded. Thus, a final multivariable model is provided.^30^ The analysis will be completed using Statistic Package Social Sciences V.29 for Windows (SPSS, Armonk, USA).

## Discussion

The estimated prevalence of LEA in male athletes ranges from 15%–70%,^1^ with some sports deemed higher risk than others due to weight categories, high energy expenditures or a focus on aesthetics.^4 5 10 11^ The prevalence within academy football players is less understood, despite evidence that energy and carbohydrate periodisation does not occur^7 17^ and the total energy expenditure is higher than age-matched non-academy players.^18^ Furthermore, players’ RMR increases with age, growth and maturation^19^ as well as demonstrating some variation across the competitive match week.^7^ The proposed study will estimate the current prevalence of LEA among male academy football players to enhance the limited literature within this population and provide data for practitioners to focus on players who might be (intentionally or unintentionally) neglecting fundamental nutritional principles.

The measurement of LEA in athletes has difficulties in validity and reliability given the self-reported and often retrospective methods of recording food intake.^16^ Further, it is often a significant burden on the athlete and/or practitioner. As such, the need to detect energy deficiency and/or symptoms has been emphasised.^8^ Indeed, the quantification of total energy expenditure using doubly labelled water has been documented in tightly controlled research studies with high internal validity.^18^ However, this is largely unrealistic for regular use across academy football due to the resources required to do such work for even a few participants. Furthermore, the service provision and finances of football academies differ across the categorisation status (1–4), with only the highest tier (category 1) required to employ a nutritionist (at least) on a part-time basis.^31^ As such, there is a need for simple, low-cost screening methods that can be distributed across multiple squads of players and identify players at risk of, or in a state of, LEA. Accordingly, the data collected from this study will be used to determine any association between LEA and various physiological and perceptual markers linked to LEA. This could result in improved screening of LEA in academy football where screening tools (eg, questionnaires, RMR_ratio_, blood samples) can be easily implemented periodically to avoid worsening symptoms and more invasive and intensive treatment methods.

## supplementary material

10.1136/bmjsem-2024-002250 online supplemental file 1

## Data Availability

Data sharing not applicable as no datasets generated and/or analysed for this study.
