# Supplementary material for: Prevalence of physiological and perceptual markers of low energy availability in male academy football players: a study protocol for a cross-sectional study
Source: BMJ Open Sport Exerc Med. 2024 Oct 7;10(4):e002250. doi: 10.1136/bmjsem-2024-002250 (PMC11459302; doi:10.1136/bmjsem-2024-002250)
Supplement: online supplemental file 1 [file bmjsem-10-4-s001.pdf]

Appendix

Appendix 1. Variables and level of measurement

Variable	Level of measurement	Collection technique	Unit of measurement
Age	Ratio	Questionnaire	Years
Body mass	Ratio	Questionnaire	Kilograms
Height	Ratio	Questionnaire	Centimetres
Occurrence of bone stress injury	Binary	Questionnaire	Yes/No
Frequency of bone stress injury	Ratio	Questionnaire	Number of events
Sex drive	Binary	Questionnaire	Low/Normal
Burnout	Binary	Questionnaire	Low/High
Training distress	Binary	Questionnaire	Low/High
Risk of eating disorder	Binary	Questionnaire	Low/High
Testosterone	Binary	Blood sample	Low/Normal
TT_3	Binary	Blood sample	Low/Normal
RMR_{ratio}	Binary	Resting metabolic rate measurement	Low/Normal
Energy intake	Nominal	3-day food diary (In-person only)<br>24h food diary (All participants)	Low/Optimal/High
Carbohydrate intake	Nominal	3-day food diary (In-person only)<br>24h food diary (All participants)	Low/Optimal/High
Protein intake	Nominal	3-day food diary (In-person only)<br>24h food diary (All participants)	Low/Optimal/High
Fat intake	Nominal	3-day food diary (In-person only)	Low/Optimal/High

24h food diary (All participants)

Note: TT_3 = total Triiodothyronine, RMR_{ratio} = Resting metabolic rate ratio.
